# Chemical and Antioxidant Charaterization of Native Corn Germplasm from Two Regions of Costa Rica: A Conservation Approach

**DOI:** 10.1155/2020/2439541

**Published:** 2020-01-27

**Authors:** Randall Syedd-León, Rafael Orozco, Víctor Álvarez, Yendry Carvajal, Gerardo Rodríguez

**Affiliations:** ^1^Department of Chemistry, Phytochemistry Laboratory, Universidad Nacional Costa Rica, Costa Rica; ^2^Department of Agricultural Sciences, Universidad Nacional Costa Rica, Costa Rica

## Abstract

The cultivation of native corn has decreased in favor of the cultivation of improved commercial corn varieties. This study seeks to evaluate the antioxidant and antibacterial potential of 36 samples of native corn germplasm from the Brunca (BR) and Chorotega (CR) regions of Costa Rica. The main parameters of comparison were the composition of antioxidant compounds, antiradical activity, and microbicidal effect. The total amount of polyphenols in the germplasm (120 mg GAE/100 g d.w.) was not related to the regions from which the samples were obtained. The overall average for antioxidant capacity was 21.20 *μ*mol TE/g d.w. Accessions from the CR region had higher antioxidant capacity. Anthocyanin content was higher in purple accessions and undetectable in white germplasm. Antioxidant capacity was statistically related to polyphenols content (*r* = 0.714, *p* < 0.01). The most promising corn accessions in terms of nutraceutical value came from the CR region.

## 1. Introduction

Corn (*Zea mays* L.) is one of the most commonly consumed foods in the world [1], and is used intensely in various ways, making it a highly important crop. Much of its current value is linked to its phenotypic characteristics and its yield in high intensity farming. Shape, color, adaptability, and above all, profitability are the most important criteria used in selecting the types of corn grown for commercial purposes. Many farms choose strains that have been genetically engineered to express desirable traits [2] that improve their commercial viability.

Native corn, on the other hand, is more often associated with traditional and subsistence agriculture. Local farmers select seeds based on empirical criteria such as the shape of corn cobs, color, and cultural aspects. Germplasm conservation has been managed by farmers without a clear government policy establishing preservation priorities. This issue should be of great concern because many farmers prefer more commercially viable strains and have discontinued harvesting local varieties, leading to their extinction. This process also leads to the loss of genetic variety as farmers abandon native varieties in favor of common monocrops.

Native varieties may be capable of providing currently undiscovered benefits, making germplasm banks necessary to preserve diversity. Research into unknown characteristics of local corn varieties may produce findings that provide economic incentives for farmers to continue using native corns. For example, if a local variety proves to have high antioxidant or antibiotic properties, its commercial viability would surely improve. In this context, Grant 0587-17 was approved by the Costa Rican government and financed by the Ministry of Science and Technology to assess the nutritional potential of Costa Rican native corn. The purpose of this project was to explore secondary metabolites, with an orientation towards finding antioxidant properties that could contribute to human health. Special emphasis was given to total polyphenols, anthocyanins, and carotenoids, which are widely known for their nutraceutical properties. Nutraceuticals are foods whose composition of secondary metabolites combines the benefits of nutrition and pharmaceutical properties, including any product derived from foods that can be used to improve or maintain health [3].

There is a wide variety of naturally occurring compounds with nutraceutical properties. The family of polyphenols is one of the most commonly studied of these compounds, due to its wide distribution and abundance in foods. The antioxidant properties of polyphenols are widely known [4], and flavonoids are the most diverse and most frequently studied polyphenol group [5]. These compounds have a diphenylpyran carbon skeleton (C_6_-C_3_-C_6′_) arranged in three rings [6].

There are many different subgroups of flavonoids, including flavanones, flavones, isoflavones, flavonols, and anthocyanidins [6]. Anthocyanidins are especially important in foods since these molecules give colors to food, such as red, purple, and blue [7] which are very much appreciated by consumers. Some studies have also found anthocyanidins with antioxidant [8–10] and antihypertensive [11] properties.

Plant polyphenols are usually considered to be nutraceuticals due to their antioxidant activity. Their other important properties include anti-inflammatory [12], antiallergic, hypotensive, and hypoglycemic effects [10]. Antioxidants can eliminate free radicals or reduce their effects. These radicals can trigger degenerative diseases (e.g., Alzheimer's, cancer) when they accumulate in an organism, causing oxidative stress [13].

Furthermore, antioxidants have been linked to antibacterial activities, which have attracted interest due to their potential for combating antibiotic-resistant bacteria. Some studies indicate that polyphenolic compounds are one of the largest groups of secondary metabolites that exhibit antimicrobial activity [6]. This bioactive effect has been explained by the number of hydroxyl groups on the phenol group, making them toxic to microorganisms. Some polyphenolic compounds have been found to be synthesized by plants in response to microbial infection, so it is no surprise that they have also been found, *in vitro*, to be an effective antimicrobial agent against a wide array of microorganisms.

The aim of this study was to chemically characterize corn germplasm in two of the most important corn-producing regions in Costa Rica, seeking to provide objective characterizations of their antioxidant profiles. The information derived from this study will be used as the basis for prioritization of agricultural programs and conservation of local corn varieties in germplasm banks. Due to the wide variations in latitude, altitude, and weather conditions between these two locations (CR is the driest part of the country and BR one of the rainiest) [14], it is expected that there might be differences in the profiles of antioxidant components.

Results of previous studies have shown that agroecological environments and climate have an influence on the composition of secondary plant metabolites [15], suggesting that the nutraceutical properties of corn might vary between these two regions. In addition, the analysis of bioactive constituents with antioxidant effects could help in the re-evaluation of native corn. This is the first study of this kind carried out in Costa Rica, although commercial or genetically engineered corn varieties have been analyzed in Mexico [16]. This study is different from previous studies in its focus on native corn varieties and its acknowledgment of the importance of the contribution of native varieties to biodiversity.

## 2. Materials and Methods

### 2.1. Chemical Reagents

6-hydroxy-2,5,7,8-tetramethyl chroman-2-carboxylic acid (Trolox), fluorescein, 2,2′-azobis (2-methylpropionamidine) dihydrochloride (AAPH), phosphate buffer, gallic acid, hydrochloric acid, sodium carbonate, and Folin-Ciocalteu reagent were purchased from Sigma-Aldrich. Cianydine-3-glucoside was purified from blackberry fruit in our Phytochemistry Laboratory (LAFIT-UNA, Costa Rica).

### 2.2. Germplasm Samples

Thirty-six corn germplasm samples were evaluated, which were provided by the germplasm bank of the Phytogenetic Resources Laboratory of the School of Agrarian Sciences of the Universidad Nacional de Costa Rica. The samples come from two geographical regions of Costa Rica, which have very different weather conditions. Of the 36 accessions, 22 are from the Chorotega Region (CR) and 14 from the Brunca Region (BR). The CR region is located in the northwest of the country and is the driest region in the country (with an average temperature of 32°C and an annual rainfall of 1800 mm), while the BR region is located in the south and has the most rainfall in the country, with an average temperature of 26°C and an annual rainfall of 3700 mm [17]. All the accessions are native materials from local producers. The colors of the accessions were not selected deliberately or randomly: they are the result of the phenotypes historically selected by local farmers and identified as native. Each accession of corn was identified by an alphanumeric code for its conservation in a germplasm bank. They were subsequently dried by lyophilization and ground in a blade mill with a 1 mm sieve. The samples were stored at 30°C until they were analyzed.

### 2.3. Optimization of Extraction Conditions

The type of solvent and the number of extractions were considered in order to determine the parameters that improved the efficiency of total polyphenol extraction in corn germplasm. Six solvent mixtures were evaluated: (A) methanol : water (7 : 3), (B) acetone : water (7 : 3), (C) acetone : methanol : water (4 : 4 : 2), (D) acetone : ethanol : water (4 : 5 : 1), (E) acetone : methanol (1 : 1), and (F) aqueous:ethanol (95%) with a solvent-corn ratio of 10 : 1. Dry and fine-powdered corn samples were put into a test tube with solvent in a sonic bath for 10 minutes and then centrifuged at 4000 rpm for 5 minutes. The samples were extracted four successive times with 2 mL of solvent, and supernatants were collected and standardized to a final volume of 10 mL. The resulting solutions were analyzed using the Folin-Ciocalteu method for total polyphenols. The number of successive extractions carried out (from one to five) was also evaluated using the best solvent obtained previously.

### 2.4. Total Polyphenol Content

The Folin-Ciocalteu Spectrophotometric method [18] was used, with some adaptations for microscale. Briefly, 250 mg of dry germplasm was placed in a test tube and extractions of polyphenols were carried out in optimal conditions with acetone : water (7 : 3) and 3 mL × 3 extractions. In a 96-well microplate 200 *μ*L of water, 15 *μ*L of Folin-Ciocalteu reagent, 30 *μ*L of extract, and 50 *μ*L of 20% carbonate sodium solution were added per well. In addition, blank wells received solvent instead of the extracted polyphenols. Aqueous gallic acid solutions from 0 to 0.112 mg/mL were used to determine the calibration curve. The plate was shaken and allowed to incubate for 20 minutes in a Synergy HT Multi-Detection Microplate Reader (BioTek Instruments) at 40°C. After incubation, absorbance was measured at 755 nm. The results were expressed as mg equivalents of gallic acid per 100 grams of dry weight germplasm (mg GAE/ 100 g d.w). All germplasm extracts were analyzed in triplicate.

### 2.5. Total Anthocyanins Content

Total anthocyanins were determined using the differential pH method [19]. The corn extracts were placed in buffer solutions of pH 1 (HCl/KCl) and pH 4.5 of sodium acetate. In a 96-well plate, 100 *μ*L of each extract and 200 *μ*L of each buffer solution were added at each pH. Absorbance (A) was measured at 510 and 700 nm (Synergy HT Multi-Detection Microplate Reader, BioTek Instruments). The absorbance was calculated according to the following equation [20].

(1) $$\begin{array}{c} A=\left({\text{Abs}}_{510}-{\text{Abs}}_{700}\right){\text{pH}}_{1}-\left({\text{Abs}}_{510}-{\text{Abs}}_{700}\right){\text{pH}}_{4.5}. \end{array}$$

A calibration curve was determined using standards of cyanidin-3-glucoside (from 0.045 mg/mL to 0.450 mg/mL). The results are reported as mg equivalents of cyanidin-3-glucoside per gram of dry germplasm (mg EC3G/g d.w.).

### 2.6. Antioxidant Activity

The Oxygen Radical Absorbance Capacity (ORAC) method was used [21] to determine antioxidant capacity. In this process, the antioxidant and the substrate (fluorescein) compete for thermally generated peroxyl radicals when an azo compound decomposes.

A Biotek 96-well microplate Synergy HT Multi-Mode fluorescence reader was used. The reaction was carried out in 75 mM phosphate buffer (pH 7.4), with a final mixing volume (reagents and sample) of 200 *μ*L per well. Solutions of 20 *μ*L of sample corn extracts and 120 *μ*L of 116.7 nM fluorescein (FL) were used, which were placed in the wells. The samples were preincubated for 15 minutes at 37°C, and 60 *μ*L of a 40 mM AAPH solution was quickly added using a multichannel pipette. The microplates were immediately placed in a fluorescence reader, and readings were made every minute for 80 minutes. The target (FL + AAPH) was prepared with phosphate buffer. Eight solutions (20 *μ*L c/u) of Trolox in phosphate buffer (100, 75, 50, 25, 12.5, 6.25, and 0 *μ*M) were used as standards for the calibration curve. The equation for the relationship between the net area under the curve (AUC) and the concentration of Trolox was determined. ORAC values were expressed as *μ*mol equivalent of Trolox per gram of dry weight (*μ*mol TE/g d.w.).

### 2.7. Antibacterial Activity Testing

Performing this test required strains isolated and purified from four pathogenic bacteria used in studies of antimicrobial sensitivity, two classified as Gram-negative (*Pseudomonas aeruginosa* and *Escherichia coli*) and two as Gram-positive (*Bacillus subtilis* and *Staphylococcus aureus*). Antimicrobial sensitivity tests were performed using the Kirby–Bauer method [22], which is also known as diffusion in agar using filter paper discs. Disks of 6 mm-diameter Whatman 42 filter paper were used, with solvent applied aseptically to each sample and allowed to dry before they were used in trials.

The concentration of each solution to be analyzed was 100 mg/mL, using distilled water or tween-80 as a solvent depending on solubility, with 3 mg applied to each disk. In accordance with the Kirby–Bauer method, petri plates sterilized by autoclaving were used. A layer of Müller Hinton 8.0 mL (4 mm thick) culture medium was placed on each plate. Once the medium solidified, the surface was inoculated with 0.1 mL of the culture of the bacterial suspension at a concentration adjusted to 100 mg/mL to coincide with a 0.5 pattern on the McFarlan scale.

The inoculum was in solutions before it was placed on the disks. 50 *μ*L of each sample with a concentration of 0.06 mg/mL in acetone : water (7 : 3) was applied to each disk. The bacteria were also evaluated using a 30 *μ*g chloramphenicol disk as the positive control and an acetone : water (7 : 3) disk as the negative control, with the latter used as the solvent to dissolve the samples to be tested. All inoculations, as well as sample applications, were performed under aseptic conditions using a laminar flow cabinet. The measurement of halos of inhibition was performed with a Vernier caliper and all tests were carried out in quadruplicate. The negative control allowed evaluation of the possibility of interference from the solvent. The halos were compared with the chloramphenicol positive control and assigned a percentage of the control value using the following formula:

(2) $$\begin{array}{c} \%\text{RPDIZ}=\frac{{\text{DIZ}}_{\text{sample}}-{\text{DIZ}}_{\text{negative control}}}{{\text{DIZ}}_{\text{positive control}}}\times 100, \end{array}$$

where RPDIZ refers to the relative percentage of the diameter of the inhibition zone and DIZ refers to the diameter of the inhibition zone. This allows establishing a relationship between the sample evaluated and the reference antibiotic using the bacterial growth inhibition zone.

### 2.8. Statistical Analysis

The results are presented as mean and standard deviations (M ± SD). Statistical analysis was performed using the statistical software IBM SPSS V 22. Normal dispersion was verified using the Kruskal–Wallis and Shapiro–Wilk tests. Homoscedasticity was determined using the Levene test. The Student's *t*-test was used for comparison between two groups, and one-way ANOVA and post-hoc Tukey tests were used for multiple comparisons. The correlation between polyphenol content and antioxidant activity was expressed using the Pearson correlation coefficient. Differences were considered significant at *p* < 0.05. The results are analyzed by regions (CR and BR). In addition, the color of corn germplasm was analyzed if it influenced the concentration of the antioxidant metabolites; for this analysis, data were subdivided into three categories: purple, yellow, and white corn.

## 3. Results and Discussion

### 3.1. Optimal Extraction Conditions

Based on the results obtained from ground corn extracts, the best solvents for the extraction of total polyphenols (TP) using the Folin–Ciocalteu method were acetone : H_2_O (7 : 3) and acetone : MeOH : H_2_O (4 : 4 : 2). For this test, the comparison of means and analysis of variance (ANOVA) using the Tukey test (*p* < 0.05) shows that both solvent mixtures have better extraction yields than the other combinations of solvents evaluated. These two solvents do not differ from each other in terms of their extractive power of phenolic compounds, and either of them could be used for this purpose. In this case, acetone : water (7 : 3) was selected because it is simpler to prepare and handle.

**Figure 1:**
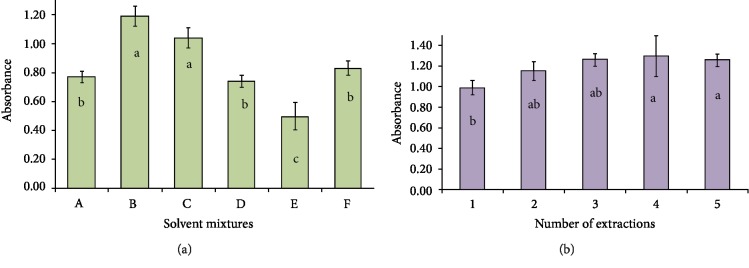
Absolute absorbance of a sample of native corn with different mixed solvent extracts (a) and progressive number of successive extractions (b), using the Folin–Ciocalteu method.

Figure 1 is a bar graph with the absolute absorbances of the samples that were extracted using each of the different mixtures of solvents. The different letters on the bars represent statistically significant differences (*p* < 0.05). TP amounts are not presented as gallic acid equivalents because absorbance is directly proportional to TP concentration. Therefore, extraction efficiency can only be determined in relative terms based on absorption.

Based on the previous test, the best solvent—acetone : H_2_O (7 : 3)—was used to determine the optimal number of successive extractions. Between one and five extractions were performed. According to the analysis of variance and the Tukey test (*p* < 0.05) from two extractions, it is not possible to significantly increase the amount of TP (Figure 1). However, three extractions were selected in all subsequent experiments, because there are no statistically significant differences in terms of the amount of extracted phenolic compounds, indicating quantitatively that three extractions from the same sample removed all phenolic compounds, and that additional extractions do not improve the amount of extracted phenolic compounds.

### 3.2. Total Polyphenol Content

Table 1 shows the concentrations of total polyphenol content (TPC) for samples from the two regions studied. A BTEC germplasm ID code indicates that a sample is from the Brunca Region (BR), while the other samples are from the Chorotega Region (CUCR).

The mean value for TPC was 120 ± 44 mg GAE/100 g d.w.; the highest value obtained was 274 (CUNA20) and the lowest 59 (CUNA04). When comparing the mean value for TPC found in this study with that of several Mexican corn varieties (173 mg GAE/100 g), it was found that the values are very similar, and that differences in TPC might be attributed to differences in varieties [16]. In another study on pigmented corn in Mexico, values ranging from 59 to 212.2 mg GAE/100 g d.w. [23] were obtained. Studies in other locations such as India found TPC values in colored corn ranging from 90.3 to 184 mg GAE/100 g d.w. [24]. A consistent result in all of these studies is that samples with intense colors have a higher TPC content. Although the results are comparable, it should be noted that the germplasm analyzed in this study is native, as opposed to other studies reported in the literature which are based on commercial varieties subject to genetic improvement.

Student's *t*-test results showed no significant differences (*t*(36) = 0.416, *p* > 0.05) between the means of native corn germplasm TPC from the different Costa Rican regions (BR and CR). Based on the study results, no definitive criteria can be established to differentiate by region since, in addition to the differences in climatic patterns and types of soil between regions, there was great genetic diversity in the germplasm used. More obvious variability occurred when considering the color of the germplasm, since tonality is indicative of the presence of terpenes and anthocyanins as secondary metabolites. The selection of cultivated corn varieties in these regions has been carried out based more on traditions than on productivity. To analyze the effect of phenotypic characteristics, the accessions were classified in terms of three colors: purple, yellow, and white.

Results of the analysis of variance show that total phenolic content in purple corn accessions was higher than that in white corn samples (*p* = 0.027). On the other hand, no significant differences between the samples of yellow and purple corn were found (*p* > 0.05), or between samples of yellow and white corn (*p* > 0.05). It can be said that corn accessions with intense colors have a higher total polyphenols content, which agrees with the results of previous studies of other foods [25].

### 3.3. Antioxidant Activity Determined Using the ORAC Assay

Table 2 shows the values of the antioxidant capacity for each corn accession analyzed. The general mean was 21.20 *μ*mol TE/g d.w., with purple accessions CUNA11, CUNA20, CUNA05, and CUNA01 having the highest values, and the results for all 36 samples ranging from 66.49 (CUNA11) to 4.81 *μ*mol TE/g d.w. (CUNA19). According to the United States Department of Agriculture (USDA), the average antioxidant activity in samples of yellow sweet corn is 7.28 *μ*mol TE/g d.w. [26], which is substantially lower than what was observed in Costa Rican naitve germplasm. On the other hand, a study with commercial species of Mexican corn showed an average value close to 50.20 *μ*mol TE/g d.w. [27]. Since the plant materials discussed here come from different locations, with differences in weather, color, and adaptive conditions, the scope of the comparisons that can be made between them is necessarily limited. With respect to other food sources, the antioxidant activity of the corn accessions is low to moderate. Berries, for example, tend to have higher values; blueberries can reach values of 274.62 *μ*mol TE/g d.w. [28]. These differences are expected due to the high concentration of pigments.

**Figure 2:**
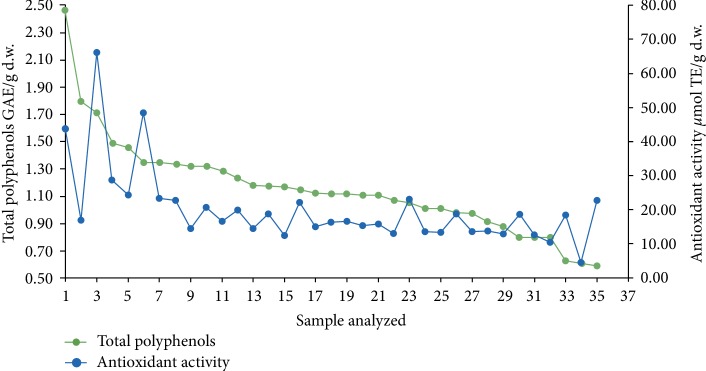
Graphical representation of the correlation between total polyphenols and antioxidant activity.

Pearson statistic was applied to the corn data set finding a correlation between the total amount of polyphenols and the antioxidant activity. The results showed that these two variables have a positive relationship (*r* = 0.714, *p* < 0.01). The magnitude of the correlation according to the Cohen coefficient is close to 0.8, which is high. This finding is similar to the results of multiple studies which have shown that polyphenols are the main secondary metabolites with antioxidant properties in many foods [29].  Figure 2 shows that there is a trend towards increased antioxidant activity with increases in total polyphenol content in most of the corn samples studied.

### 3.4. Total Anthocyanin Content

The total amount of anthocyanins determined using the differential pH method was reported as cyanidin-3-glucoside milligram equivalents per gram of dry weight (mg EC3G/g d.w.). Anthocyanins are flavonoid compounds with colors ranging from blue to purple [30]. Therefore, the analyses were performed using only colored (yellow and purple) samples of the collected corn germplasm. Some of the yellow accessions did not contain anthocyanins, and results for these accessions do not appear in Table 3.

The values obtained ranged from 3.96 mg EC3G/g d.w. (CUNA01) to 0.44 mg EC3G/g d.w. (BTEC15), with an average value of 1.97 mg EC3G/g d.w. In comparative terms, these values are high. In Mexico, a native blue corn variety was reported to have an anthocyanin content of 0.96 mg EC3G/g d.w. [30], considerably lower than the value found for the native Costa Rican accessions analyzed here. However, there are genetically improved purple varieties in the market that over-express anthocyanin contents. The Thai variety KPSC 901 contains up to 3.97 mg EC3G/g d.w. [31]. Since this variety is considered to have a high anthocyanin content, the native CUNA01 accession is very appealing in this regard, since its anthocyanin content is nearly as high.

Some fruits typically recognized as having high anthocyanin contents, such as cranberries, can reach values close to 10.5 mg EC3G/g d.w. [29], considerably higher than the value reported for corn. While these foods may contain more anthocyanins, this advantage is offset by their price, since corn is much more economical to produce. As a result of its price, as well as the energy input that it requires, corn is consumed more widely at a global level.

Some native corn accessions may constitute a source of natural pigments for the food industry, due to their anthocyanin content. The added value of pigments based on anthocyanins is that they provide antioxidant capacity, are easily incorporated into the aqueous media of food [32], and can be prepared in many different ways.

The quantity of anthocyanins in the Costa Rican samples is directly related to the total content of polyphenols (*r* = 0.59, *p* < 0.05) and to their antioxidant capacity (*r* = 0.528, *p* < 0.05). This finding is consistent with other descriptions in the literature since anthocyanins are the most important group of colored polyphenols in foods in terms of antioxidant capacity.

The results of antibiograms are shown in Table 4. Even though tests were conducted for all the samples, results are only shown for cases in which at least one of the microorganisms was inhibited; only 12 extracts of the 36 accessions showed some type of inhibitory activity. In general, the inhibitory responses vary among the bacteria tested. Results of the antibiograms obtained with extracts of corn accessions showed very low antimicrobial activity in almost all cases. None of the extracts of the accessions showed a strong response against the growth of all four of the microorganisms tested. Three of the accessions (CUNA32, CUNA38, and CUNA11) inhibited growth in two of the four bacteria, while the remaining samples were active against only one of them. In the case of accessions that are active against bacterial growth, it said that Gram-positive bacteria are more susceptible than Gram-negative bacteria to antimicrobial agents present in the corn germplasm. The data indicate that the microorganism *S. aureus* is most sensitive to corn germplasm among the four bacteria analyzed, while *E. coli* is the least sensitive. Part of the resistance found in Gram-negative bacteria might be due to the fact that they have cell walls with several complex layers, and have external proteins and lipopolysaccharides in an extra layer of the peptidoglycan, while Gram-positive bacteria have only a single layer [33]. Antimicrobial activity may be the result of many different secondary metabolites, each of which is an adaptive response of plants against possible attacks by pathogens. There is no evidence that polyphenols are the type of compound that may be responsible for microbiological activity. The result of Pearson correlation tests (*p* < 0, 0.05) show that there is no significant relationship between biocidal activity and total polyphenol content or the concentration of anthocyanins. It should be noted that because there were few samples in which the zone of inhibition could be measured, the results are not statistically robust. However, corn displayed inhibitory effects on the growth of *Salmonella enteritidis* (ATCC13076), *Staphylococcus aureus* (ATCC 6538), and *Candida albicans* (ATCC 10231), while no corn extract produced such effects on *Escherichia coli* (ATCC 11775) [11]. These findings are consistent with the results of this study, since *E. coli* showed the least response to antibiotic activity. Actually, *E. coli* lives in mammalian digestive tracts without major effects. Even though their presence can be hazardous to their hosts, the prescription of antibiotics for strong/healthy/muscular people is often avoided because the bacteria can mutate and generate resistance to antibiotics during prolonged treatments [34].

## 4. Conclusion

Native Costa Rican corn germplasms are an important source of polyphenols, making these crops valuable food resources. Although the concentration of polyphenols in corn is lower than that found in other foods such as berries, consumption of corn is much higher. This study determined that there was no significant difference in the abundance of polyphenols in accessions from the Brunca or Chorotega regions of Costa Rica, although native varieties were found to be better sources of antioxidants than common commercial varieties previously described.

Antioxidant activity was directly related to the total amount of polyphenols in the accessions analyzed in this study. Purple accessions have relatively high values of anthocyanins, above those of the most common commercial corn discussed in the literature. There is statistical support for the conclusion that samples with higher anthocyanin content have greater antioxidant activity, making accessions of purple corn more valuable as food sources. Native corn cultivated in the Chorotega region has a higher nutraceutical value in terms of anthocyanin content and antioxidant activity. In terms of total polyphenols, there are no significant differences between accessions from the two regions, but in general terms, corn from the Chorotega region was found to be a more valuable food resource, particularly in the cases of accessions CUNA01, CUNA20, and CUNA11. Germplasms of corn from the Chorotega and Brunca regions have very low microbicidal effects against the growth of *E. coli, P. aeruginosa, S. aureus*, and *B. subtilis*. Gram-positive bacteria are more susceptible to phytochemicals from the accessions analyzed; the microorganism *S. aureus* is more susceptible to the greatest number of corn accessions, while *E. coli* is the most resistant. The total content of polyphenolic compounds showed no significant relationship with biocidal activity, which must, therefore, be the result of secondary metabolites of a different chemical nature.

## Figures and Tables

**Table 1:** Total polyphenol content present in accessions of native corn from the Brunca and Chorotega regions, using the Folin–Ciocalteu method.

Germplasm ID	Color	mg GAE /100 g d.w.	Germplasm ID	Color	mg GAE/100 g d.w.
BTEC02	White	97 ± 6.0^d–i^	CUNA05	Purple	135 ± 15^b–e^
BTEC03	White	107 ± 7.0^d–i^	CUNA08	White	80 ± 7.0^f–i^
BTEC04	White	101 ± 8.0^d–i^	CUNA11	Purple	172 ± 19^bc^
BTEC05	White	102 ± 1.0^d–h^	CUNA12	Purple	88 ± 5.0^e–i^
BTEC06	White	112 ± 8.0^d–i^	CUNA14	White	98 ± 4.0^d–i^
BTEC07	Yellow	118 ± 8.0^def^	CUNA15	Purple	92 ± 11^e–i^
BTEC08	White	180 ± 9.0^b^	CUNA17	White	63 ± 3.0^ghi^
BTEC09	Yellow	132 ± 22^b–f^	CUNA19	Yellow	61 ± 6.0^hi^
BTEC10	Yellow	129 ± 8.0^b–f^	CUNA20	Purple	274 ± 11^a^
BTEC11	White	111 ± 9.0^d–i^	CUNA24	Purple	134 ± 0.1^b–e^
BTEC12	Yellow	118 ± 5.0^def^	CUNA25	White	80 ± 5.0^f–i^
BTEC13	White	80 ± 1.0^b–e^	CUNA29	Yellow	124 ± 0.1^c–f^
BTEC14	Yellow	132 ± 7.0^b–f^	CUNA31	Yellow	112 ± 5.0^d–i^
BTEC15	Yellow	117 ± 6.0^def^	CUNA32	Yellow	112 ± 2.0^d–i^
CUCR03	Purple	110 ± 1.0^d–i^	CUNA35	Purple	146 ± 14^bcd^
CUCR05	Purple	105 ± 6.0^d–i^	CUNA38	White	115 ± 3.0^d–g^
CUNA01	Purple	245 ± 12^a^	CUNA39	White	135 ± 6.0^b–e^
CUNA04	Yellow	59 ± 1.0^i^	CUNA44	Yellow	149 ± 2.0^bcd^

Data are expressed as mean ± SD. BTEC comes from the Brunca region, while the rest are from the Chorotega region. Different letters indicate significant differences (*p* < 0.05) using the Tukey test in the total set of accessions.

**Table 2:** Characterization of antioxidant activity of corn accessions from the Chorotega and Brunca regions, obtained using the ORAC method.

Germplasm	*μ*mol TE/g d.w.	Germplasm	*μ*mol TE/g d.w.
CUNA11	66.49 ± 0.51^a^	BTEC10	16.93 ± 2.69^cde^
CUNA20	55.41 ± 8.03^ab^	CUNA31	16.71 ± 2.73^def^
CUNA05	48.06 ± 1.93^b^	CUNA32	16.49 ± 1.83^def^
CUNA01	43.72 ± 1.25^b^	CUCR03	15.85 ± 0.07^def^
CUNA44	28.68 ± 2.38^c^	BTEC11	15.50 ± 0.48^def^
CUNA35	24.64 ± 0.28^cd^	BTEC06	15.04 ± 3.19^def^
CUNA39	23.46 ± 1.65^cd^	BTEC07	14.66 ± 3.27^def^
CUNA24	23.18 ± 0.56^cd^	BTEC14	14.55 ± 2.26^def^
CUCR05	23.09 ± 0.52^cd^	CUNA15	13.92 ± 0.41^def^
CUNA04	22.88 ± 6.42^cd^	BTEC02	13.72 ± 2.30^def^
CUNA38	22.42 ± 3.61^cde^	BTEC05	13.55 ± 2.67^def^
BTEC09	20.96 ± 6.36^cde^	BTEC04	13.43 ± 2.43^def^
CUNA29	19.95 ± 2.10^cde^	CUNA12	13.18 ± 4.92^def^
BTEC12	19.04 ± 1.88^cde^	BTEC03	13.13 ± 2.40^def^
BTEC13	18.72 ± 2.76^cde^	CUNA25	12.97 ± 2.32^def^
CUNA14	18.69 ± 0.38^cde^	BTEC15	12.90 ± 3.55^def^
CUNA17	18.53 ± 1.57^cde^	CUNA08	10.72 ± 0.28^ef^
BTEC08	17.11 ± 2.99^cde^	CUNA19	4.81 ± 0.61^f^

Data are expressed as mean ± SD. BTEC samples come from the Brunca region, while the rest are from the Chorotega region. Different letters indicate significant differences (*p* < 0.05) in Tukey test in the total set of accessions.

**Table 3:** Concentration of total anthocyanins in accessions of native corn from the Brunca and Chorotega regions, obtained using the differential pH method.

Germplasm	Color	mg E C3G/g d.w.
CUNA01	Purple	3.962 ± 0.068^a^
CUNA31	Yellow	3.072 ± 0.264^b^
CUNA20	Purple	2.803 ± 0.158^bc^
CUNA11	Purple	2.738 ± 0.254^bcd^
CUNA32	Yellow	2.670 ± 0.386^bcd^
CUNA44	Yellow	2.608 ± 0.118^bcd^
CUCR03	Purple	2.597 ± 0.063^bcd^
CUNA35	Purple	2.597 ± 0.181^bcd^
CUNA29	Yellow	2.382 ± 0.239^cde^
CUNA24	Purple	2.253 ± 0.097^de^
CUNA05	Purple	2.016 ± 0.229^e^
CUCR05	Purple	1.474 ± 0.062^f^
CUNA12	Purple	1.161 ± 0.081^f^
CUNA15	Purple	1.126 ± ± 0.211^f^
BTEC08	White	0.560 ± 0.449^g^
CUNA19	Yellow	0.555 ± 0.081^g^
CUNA04	Yellow	0.492 ± 0.208^g^
BTEC15	Yellow	0.435 ± 0.434^g^

Data are expressed as mean ± SD. BTEC accessions are from the Brunca region, while the rest are from the Chorotega region. Different letters indicate significant differences (*p* < 0.05) found using the Tukey test on the total set of accessions.

**Table 4:** Antimicrobial activity expressed as a relative percentage of the diameter of the inhibition zone (mm) of corn germplasm using the Kirby–Bauer method.

Germplasm	Gram-positive bacteria		Gram-negative bacteria	
	*S. aureus*	*B. subtilis*	*E. coli*	*P. aeruginosa*
	RPDIZ (%)	RPDIZ (%)	RPDIZ (%)	RPDIZ (%)
CUNA32	43.18 ± 3.90^ab^	75.67 ± 8.94^a^	—	—
CUNA38	46.47 ± 1.49^ab^	70.19 ± 13.02^a^	—	—
BTEC07	52.98 ± 13.64^ab^	—	—	—
BTEC11	46.77 ± 0.71^ab^	—	—	—
UCR05	60.29 ± 3.04^a^	—	—	—
CUNA11	38.92 ± 6.67^ab^	—	36.34 ± 17.30	—
CUNA19	46.93 ± 13.56^ab^	—	—	—
CUNA24	34.42 ± 3.56^b^	—	—	—
CUNA35	—	76.26 ± 7.09^a^	—	—
BTEC06	—	—	—	43.71 ± 5.29^a^
CUNA04	—	—	—	43.35 ± 2.76^a^
CUNA44	—	—	—	30.92 ± 0.15^b^

Control (+)	100%	100%	100%	100%

Control (−)	0%	0%	0%	0%

Data are expressed as mean ± SD. Different letters in the “Tukey HSD” columns indicate significant differences in each column according to the Tukey Test (*p* < 0.05). Tukey HSD: Honest Significant Difference. SD: standard deviation.

## Data Availability

The data used to support the findings of this study are available from the corresponding author upon request.
